# Large language models in medicine: A review of current clinical trials across healthcare applications

**DOI:** 10.1371/journal.pdig.0000662

**Published:** 2024-11-19

**Authors:** Mahmud Omar, Girish N. Nadkarni, Eyal Klang, Benjamin S. Glicksberg

**Affiliations:** 1 Maccabi Health Services, Israel; 2 The Division of Data-Driven and Digital Medicine (D3M), Icahn School of Medicine at Mount Sinai, New York, United States of America; 3 The Charles Bronfman Institute for Personalized Medicine, Icahn School of Medicine at Mount Sinai, New York, United States of America; Washington University in Saint Louis, UNITED STATES OF AMERICA

## Abstract

This review analyzes current clinical trials investigating large language models’ (LLMs) applications in healthcare. We identified 27 trials (5 published and 22 ongoing) across 4 main clinical applications: patient care, data handling, decision support, and research assistance. Our analysis reveals diverse LLM uses, from clinical documentation to medical decision-making. Published trials show promise but highlight accuracy concerns. Ongoing studies explore novel applications like patient education and informed consent. Most trials occur in the United States of America and China. We discuss the challenges of evaluating rapidly evolving LLMs through clinical trials and identify gaps in current research. This review aims to inform future studies and guide the integration of LLMs into clinical practice.

## Introduction

Large language models (LLMs) are artificial intelligence (AI) systems trained on vast text data to understand and generate human-like language [1]. This technology emerged as a particularly important recent innovation and are likewise being evaluated in medical practice and research [1–4]. There have been many studies that show promise for LLMs, such as GPT (generative pre-trained transformer), BERT (bidirectional encoder representations from transformers), in healthcare for patient interaction, administrative tasks, data analysis [5–7], and beyond. However, LLMs can produce inaccurate information and have been shown to propagate bias raising concerns about their use in clinical settings [1,8].

Like with all potential machine learning-based tools, clinical trials are needed to evaluate the effectiveness and safety of LLMs in real-world medical applications. These trials are research studies that assess new interventions, including technologies like LLMs, in clinical workflows [9]. These trials follow standardized protocols and are essential for validating healthcare innovations before widespread adoption [10].

Recent papers on LLMs in healthcare often present conflicting results [11]. Some clinical trials have shown some possible disparities compared to nonclinical studies. For example, while Liu and colleagues summarized that GPT is effective for clinical documentation [12] and Barrak and colleagues reported positive perceptions among pediatric emergency medicine attendings [13], a randomized controlled trial (RCT) by Baker and colleagues revealed errors in 36% of documents [14]. We will focus solely on registered clinical trials to maintain coherence, despite the valuable evidence some nonclinical trials offer regarding the potential benefits, biases, and inaccuracies of LLMs [1,8].

This review analyzes registered clinical trials exploring LLM applications in medicine. We focus on LLMs due to the growing research interest in their potential impact on healthcare [1]. Our analysis covers trial designs, applications, and outcomes to identify trends and gaps in current LLM research. This review aims to inform future studies and guide the integration of LLMs into clinical practice and research.

## Methodology

### Search strategy and selection criteria

We systematically screened publications from January 2018, when the first public LLM debuted. Our search terms included “clinical trials,” “Large Language Models,” “LLMs,” “GPT,” and “BERT.” We used PubMed, Scopus, Embase, ClinicalTrials.gov, and the International Clinical Trials Registry Platform (ICTRP) to find published and ongoing research. We used the Rayyan web application [15], a tool designed for streamlined screening of academic papers. Our review follows the PRISMA extension for scoping reviews guidelines [16]. The Boolean strings used for database searches are in the Supporting information S1 Text.

### Inclusion and exclusion criteria

We included registered clinical trials and RCTs that evaluated LLMs in clinical practice or research. This encompassed both published trials and registered, unpublished clinical trials. We selected trials where LLMs were the primary intervention. We define LLMs as neural network-based models trained on large text data sets to generate human-like text [1]. We did not include trials using linear models (e.g., logistic regression) or other machine learning models (e.g., decision trees, non-LLM neural networks).

### Screening and data extraction

The initial screening of titles and abstracts was conducted by 2 independent researchers, MO and EK. Following the initial screening, full-text articles and records of registered and currently running clinical trials deemed potentially eligible were retrieved and assessed. Data extraction was performed by one reviewer (MO) and subsequently verified by a second reviewer (EK or BG). Any discrepancies encountered during the data extraction phase were resolved through discussion among the reviewers.

### Overview of the included trials and applications

**Fig 1:**
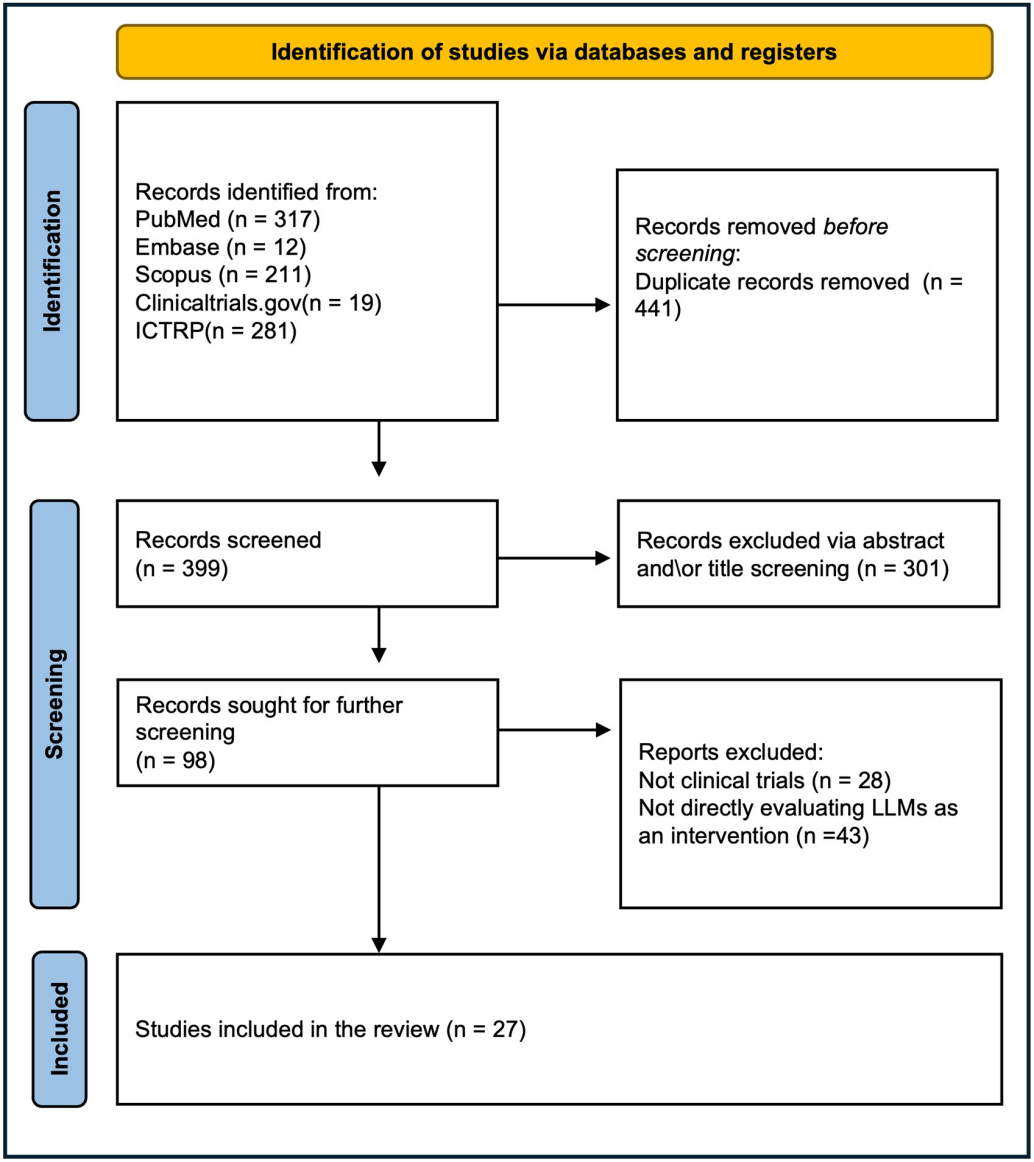
PRISMA flowchart.

Our review encompasses 27–5 published and 22 ongoing—clinical trials employing LLMs across various healthcare applications (Tables 1–3) [14,17–42]. Fig 1 represents the screening process using the PRISMA flowchart.

**Table 1:** A summary of the published clinical trials.

Author	Year	Country	Sample size	Model	Category	Objective and results
**Baker et al.**	2024	USA	11	GPT-4	**Data Handling**	**Objective: Assess ChatGPT’s ability to assist in clinical documentation.** Results: ChatGPT produced longer, higher-quality histories but included some erroneous information.
**Civettini et al.**	2024	Italy	6	GPT-4, PaLm 2, Llama-2 13b, and Llama-2 70b	**Decision and Diagnostics Aid**	**Objective: Evaluate LLMs in hematopoietic stem cell transplantation decision-making.** Results: LLMs showed moderate agreement with expert consensus but were outperformed by medical residents.
**Deveci et al.**	2023	Denmark	36	GPT-4	**Research Assistance**	**Objective: Compare the effectiveness of ChatGPT-4 and humans in writing scientific cover letters.** Results: No significant differences in impression, readability, or detail; GPT-4 was comparable to humans.
**Lawrence et al.**	2024	USA	40 (compared 20 AI-generated abstracts with 20 human-written abstracts)	GPT-3	**Research Assistance**	**Objective: Compare AI-generated and human-written abstracts for arthroplasty literature.** Results: AI and human texts were similar in quality, with AI texts presenting authorship discernibility challenges.
**Bitar et al.**	2022	Saudi Arabia	386	BERT	**Patient Care**	**Objective: Use BERT for summarizing HPV information texts**. Results: BERT-generated summaries were slightly less effective than full texts but showed potential in educational contexts.

GPT-4, generative pre-trained transformer; BERT, bidirectional encoder representations from transformers; PaLm 2, pathways language model 2; Llama-2 13b and Llama-2 70b, variants of the Llama language model.

**Table 2:** A summary of the ongoing clinical trials.

ID	Country	Target sample size	Model	Category	Study objective and outcomes
**NCT06346496**	China	657	LLM-based AI Dialogue Bot	Patient care	Evaluate LLM’s effect on anxiety, mood, and depression in young adults over 28 days. Outcomes will be measured at multiple time frames.
**ChiCTR2400081938**	China	1,000	GPT	Patient care	Compare ChatGPT’s impact on hypertension management with traditional online consultations. Focus on blood pressure compliance.
**NCT06321328**	Turkey	398	GPT-4	Decision and diagnostics aid	Assess GPT-4’s ability to predict postoperative intensive care needs. Evaluate anesthesia method recommendations.
**DRKS00033775**	Germany	600	GPT	Patient care	Determine if medical laypersons make better urgency decisions using a symptom checker or ChatGPT. Measure accuracy of decisions.
**NCT06276049**	China	103	GPT	Decision and diagnostics aid	Study the impact of ChatGPT on self-directed learning among medical students. Measure changes in learning scales and critical thinking.
**NCT06263855**	USA	1,015	CURE	Data handling	Investigate if LLM-assisted writing of discharge summaries improves care delivery. Focus on rate of patient accrual.
**NCT06247475**	Taiwan	120	GPT-3.5, GPT-4	Decision and diagnostics aid	Simulate virtual consultations, assess correctness of LLM responses and satisfaction.
**NCT06229379**	China	84	Digital twin patient	Patient care	Evaluate the use of a Digital Twin Patient in medical education to enhance clinical questioning skills.
**NCT06208423**	USA	50	GPT-4	Decision and diagnostics aid	Examine the use of GPT-4 in clinical management case discussions. Outcomes include management reasoning and time efficiency.
**ChiCTR2300078274**	China	60	GPT-4	Patient care	Explore the use of ChatGPT for informed consent in knee arthroplasty. Assess anxiety relief and patient satisfaction.
**NCT06157944**	USA	50	GPT-4	Decision and diagnostics aid	Randomize physicians to diagnose with or without GPT-4 assistance. Measure diagnostic accuracy and time.
**DRKS00032895**	Germany	200	GPT-4	Decision and diagnostics aid	Test accuracy of laypeople, symptom checkers, and LLMs with patient-generated vignettes. Measure decision accuracy.
**NCT06002425**	Germany, Italy, China, USA	400	GPT	Decision and diagnostics aid	Evaluate ChatGPT’s effectiveness in recommending treatment plans for gastrointestinal cancers. Measure influence on treatment plans.
**NCT05963802**	Canada	26	GPT	Research assistance	Assess AI’s usability and efficacy in health sciences training. Outcomes include system usability and student perception.
**NCT05945004**	USA	120	GPT-4	Data handling	Compare the efficacy of ChatGPT versus humans in writing preoperative visit sheets. Measure clinical use satisfaction.
**NCT06009783**	Canada	40	GPT	Patient care	Evaluate ChatGPT in pre-vasectomy counseling. Focus on consultation efficiency and patient satisfaction.
**ChiCTR2300071774**	China	116	GPT	Data handling	Use ChatGPT as a learning aid in orthopedics. Measure exam scores and learning style impacts.
**JPRN-UMIN000050398**	China	40	GPT	Patient care	Test anesthesia explanations by ChatGPT versus anesthesiologists. Assess patient satisfaction and understanding.
**NCT05231174**	China	535	Not specified	Decision and diagnostics Aid	Use an LLM to assist in detecting diabetic retinopathy. Measure AUROC of the self-evaluation tool.
**NCT06015178**	China	60	Not specified	Research assistance	Enhance medical researchers’ self-learning with an intelligent language model. Measure task completion times.
**NCT04246346**	China	660	Not specified	Patient care	Enhance informed choice in cataract surgery with a chatbot. Measure proportion of informed decisions made by patients.
**NCT05789901**	Canada	400	MARVIN chatbot	Patient care	Provide varied health condition information via chatbot. Measure acceptability, usability, and intervention appropriateness.

LLM, large language model; AI, artificial intelligence, GPT, generative pre-trained transformer; CURE, checker for unvalidated response errors; SUS, system usability scale; AUROC, area under the receiver operating characteristic; USA, United States of America; RCT, randomized controlled trial; NCT, National Clinical Trial number; ChiCTR, Chinese Clinical Trial Registry; JPRN, Japan Pharmaceutical Information Center—Clinical Trials Information; DRKS, German Clinical Trials Register.

**Table 3:** LLM applications in healthcare scenarios.

The category of the study application	The number of studies that evaluated this application
Patient care	11
Decision and diagnostic aid	8
Research assistance	4
Data handling	4

Despite the diverse and sometimes unique applications of these trials, they can broadly be grouped into 4 categories: Patient Care (11 trials), Data Handling (4 trials), Decision and Diagnostics Aid (8 trials), and Research Assistance (4 trials).

Patient Care encompasses any use-case directly oriented towards patients, such as management or patient education. Data Handling focuses on applications involving data analysis, storage, and related activities. Decision and Diagnostics Aid covers the diagnosis and detection of diseases. Research Assistance pertains to applications related to proofreading, writing, or reviewing research materials.

Concerning the models assessed, all published trials identified the specific models evaluated. Three trials focused on GPT-4, one on GPT-3 alongside other models such as Llama and PALM, and one on BERT. In the ongoing trials, 4 did not specify which LLM would be used. Fifteen trials employed various iterations of GPT, with 7 specifying the use of GPT-4. The remaining trials involved other models, including digital twin and BERT (S1 and S2 Tables).

## Published clinical trials

It is important to note that not all clinical trial results are published. Often, only trials that confirm researchers’ assumptions or show significant results are published [43].

**Fig 2:**
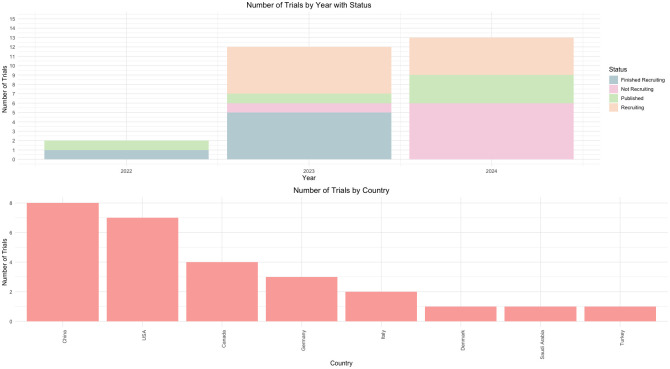
Trends in the clinical trials investigating LLMs—Years and countries. The years in the above figure represents either the year of publication (in the published trials) or the year of registration (in the running trials). The phrase “Not recruiting” conveys trials that are registered but have not started recruiting.

The review identified 5 published clinical trials (Tables 1 and 2) which examined different applications of LLMs in healthcare: 1 focused on patient care [20], another on data handling [14], 1 on decision and diagnostic aid [18], and 2 on research assistance [17,19]. While the trials were broadly categorized, they encompass a wide range of specific applications that can vary significantly even within the same category. Specifically, these trials explored applications in clinical documentation [14], medical decision-making [18], and patient knowledge enhancement tools [20]. These trials are conducted in many countries, including USA, Italy, Denmark, and Saudi Arabia, employing models such as GPT-4 and BERT (Fig 2).

In terms of outcomes, collectively, these trials showcase where LLMs exhibit promising capabilities, albeit with some limitations. For example, Baker and colleagues explored the utility of GPT-4 in enhancing clinical documentation quality in a single-center trial in the USA with 11 participants. They reported that while GPT-4 produced more detailed patient histories, inaccuracies were noted in 36% of cases. In parallel, several ongoing trials are delving into similar realms of LLM application in medical documentation [14]. Notably, NCT06263855, also in the USA, is targeting a larger cohort of 1,015 participants, examining whether LLM-assisted writing of discharge summaries can improve care delivery. Meanwhile, ChiCTR2300078274 in China is exploring the use of ChatGPT for informed consent in knee arthroplasty, and NCT05945004 is comparing the efficacy of ChatGPT with human efforts in drafting preoperative visit sheets.

In Italy, Civettini and colleagues evaluated LLMs for decision-making in hematopoietic stem cell transplantation in a multicenter Italian study with 6 participants [18]. They tested GPT-4, PaLm 2, and 2 versions of Llama-2 against medical residents and expert consensus. LLMs achieved a median overall agreement of 58.8% with expert consensus, with kappa values between 0.3 and 0.61. Residents outperformed LLMs, showing 76.5% agreement and kappa values of 0.4 to 0.8. No current trials specifically examine LLMs in stem cell transplantation decisions. However, studies like DRKS00033775 (Germany) and NCT06157944 (USA) are exploring LLMs as diagnostic aids for doctors, focusing on their assistive role rather than standalone capabilities.

Deveci assessed the capabilities of GPT-4 in writing cover letters for scientific submissions in a single-center study in Denmark involving 36 participants [17]. The findings showed that GPT-4’s letters were comparable in impression and readability to those written by humans, with slight variances in meeting specific criteria.

Lawrence and colleagues revealed that AI-generated arthroplasty literature was indistinguishable in authorship discernibility from human-written texts, though it fell short in perceived quality [19]. Interestingly, there are no ongoing trials that are directly investigating the writing aspects of research assistance capabilities of LLMs.

Lastly, Bitar’s study (2022) in Saudi Arabia with 386 participants employed BERT to summarize texts about HPV, aiming to enhance knowledge dissemination [20]. The BERT-generated summaries were effective, though slightly less so than full texts. In a related vein, NCT05789901 is evaluating the provision of health condition information via a chatbot. Although other ongoing trials are investigating the capabilities of LLMs to provide accurate and usable medical information, their focus is primarily on healthcare experts rather than patients. For example, NCT05963802 in Canada are examining the AI’s usability and efficacy in health sciences training, and NCT06015178 in China is focused on enhancing medical researchers’ self-learning abilities.

## Ongoing trials

Our review of clinical trial registries uncovered 22 registered trials currently exploring the applications of LLMs in healthcare (Table 3). These studies, registered between 2023 and 2024, are in various stages, with 9 actively recruiting participants (41% of the total), 7 have not yet started, and the remaining 6 are either ongoing or nearing completion. The trials are primarily conducted in China and the USA, accounting for 14 of the 22 trials (64%). Additional countries include Germany, Italy, and Canada (Fig 2). Sample sizes among these trials vary significantly, ranging from fewer than 100 to over 1,000 participants.

These trials predominantly utilize different models of GPT. For example, Dong and colleagues’ multicenter trial involves over 1,000 participants to assess LLMs’ effectiveness in providing decision support for gastrointestinal cancer treatments, assessing the practical utility of LLMs in complex medical decision-making scenarios.

Prominent use cases for LLMs in these trials include clinical decision support and enhancing patient care. For example, the study by Dong and colleagues evaluated LLMs in providing decision support for gastrointestinal cancer treatments, while Bitar and colleagues focused on using LLMs to enhance patient education about HPV. However, some trials featured unique and intriguing applications that highlight the innovative potential of LLMs. For example, Shalong and colleagues are investigating how a custom GPT can support self-directed learning among medical students, an innovative approach in medical education.

Another distinctive application by Zheng and colleagues explores using LLMs to improve informed decision-making during cataract surgery consultations, aiming to enhance patient understanding and satisfaction. Additionally, Yao and colleagues are assessing the impact of LLMs on discharge summary writing, which could revolutionize administrative tasks in healthcare by improving efficiency and accuracy.

The distribution of these trials provides insights into the current state of LLM integration into clinical research. There is a clear emphasis on employing LLMs for diagnostic and decision support, alongside an interest in using these models to augment medical education and patient care. Notably, the USA and China emerge as leading contributors to this field, signaling a drive towards AI adoption in their healthcare systems.

## Discussion

Our review identified 22 ongoing and 5 published clinical trials evaluating LLMs in medicine. These trials aim to assess the effectiveness and safety of LLMs in real-world healthcare settings. The accuracy and reliability standards for LLM use in clinical practice remain undefined. Future research should establish clear acceptance criteria for LLMs in various medical applications.

The reviewed trials cover diverse applications, from clinical documentation to medical decision-making. For example, NCT06263855 examines LLM-assisted writing of discharge summaries with over 1,000 participants. However, only 3 out of 27 trials focus on direct patient education interaction. For clarity, this means that the evaluated models could be used and interacted with directly by patients for educational purposes. This suggests a need for more research in these areas to fully explore LLMs’ potential in improving patient outcomes.

A key barrier to conducting clinical trials on direct patient interactions with LLMs is the lack of HIPAA compliance in many of these technologies [44]. HIPAA regulations protect patient privacy and secure health information [45]. Without proper compliance, LLMs cannot be legally or ethically used to handle patient data in clinical settings [45]. This challenge needs to be addressed to enable more comprehensive research on LLMs in direct patient care.

Trial scales vary significantly. Some, like NCT06157944, are large multicenter studies investigating LLMs in diagnostic processes. Others are smaller, single-center studies. This variation limits our ability to draw generalized conclusions about LLM efficacy and safety across different clinical settings. More large-scale, multicenter trials could provide more robust evidence.

Many trials (15 out of 27) explore multiple LLM applications within a single study. For example, NCT05963802 in Canada evaluates LLMs in various aspects of health sciences training. While this approach offers a broad view of LLM capabilities, it may lack depth in specific applications. Targeted studies focusing on individual clinical tasks could provide more detailed insights into optimizing LLMs for specific healthcare functions.

An important question that should be discussed is how to design larger clinical trials, which typically require long durations and significant efforts [10], to evaluate LLMs that are rapidly evolving [1,3]. The current evidence suggests that newer models, developed in a very short time frame, have shown significantly better results across almost all use cases [46–50].

This raises an important open issue: how can we design and execute effective, fast-paced clinical trials that can keep up with this swiftly advancing field, while maintaining the rigorous standards of robust investigative tools that are trusted and proven? Such a balance is essential to ensure that as LLMs develop, they are evaluated thoroughly and accurately, allowing healthcare to benefit from the latest advancements without sacrificing reliability.

The current state of clinical trials often lacks specificity regarding the LLMs used, with many focusing on various iterations of GPT. GPT is widely utilized and studied [51], and research consistently shows performance variations among its versions, such as GPT-3.5 and GPT-4 [48]. These performance differences are narrowing in more advanced models, illustrated by minor discrepancies between GPT-4 and its newer counterpart, GPT-4o [52]. This trend underscores the rapid development within the field, necessitating thorough and precise evaluation. Moreover, the field boasts a diverse array of models differing in parameters, design, and overall performance and safety [53].

Our review has several limitations. The databases selected might miss relevant studies not included in those sources. We included many yet-to-be-published studies to reflect the field’s rapid advancement, but this introduces uncertainty about the actual outcomes. Additionally, by excluding non-randomized studies and other AI models, we might have missed broader applications and impacts of AI in healthcare. As this field evolves quickly, there’s also a risk that the most recent studies were not included. Some registered clinical trials may not use robust, protocol-coherent designs typically accepted for clinical trials. However, we carefully screened each included study to ensure their designs are consistent with evaluating LLM interventions on human subjects.

In conclusion, LLMs offers a promise in healthcare but also requires more careful investigation and validation. Future directions should include expanding research into underexplored areas such as direct patient care and education, designing larger multicenter trials, and balancing broad-based LLM applications with targeted studies that probe defined and specific clinical tasks. Future trials should focus particularly on direct patient interactions and education—areas ripe for development but currently underexplored. Effective integration into clinical practice will require standardized protocols that ensure these models enhance, rather than compromise, the quality of care.

## Supporting information

S1 Text Full Booleans for the systematic literature search. (DOCX)

S1 Table A detailed summary of the included ongoing clinical trials. (DOCX)

S2 Table A detailed summary of the included published clinical trials. (DOCX)
