# Supplementary material for: Large language models in medicine: A review of current clinical trials across healthcare applications
Source: PLOS Digit Health. 2024 Nov 19;3(11):e0000662. doi: 10.1371/journal.pdig.0000662 (PMC11575759; doi:10.1371/journal.pdig.0000662)
Supplement: S1 Text — (DOCX) [file pdig.0000662.s001.docx]

**S1 Text**

**Validation of Large Language Models: A Review of Current Clinical Trials Across Healthcare Applications**

Mahmud Omar^1^, Benjamin S. Glicksberg^2,3^, Girish N. Nadkarni^2,3^, Eyal Klang^2,3^.

^1^ Tel-Aviv University, Faculty of Medicine.

^2^ Hasso Plattner Institute for Digital Health at Mount Sinai, Icahn School of Medicine at Mount Sinai, New York, NY, USA.

^3^ The Division of Data-Driven and Digital Medicine (D3M), Icahn School of Medicine at Mount Sinai, New York, NY, USA.

**Search strings across the different Databases.**

**PubMed**

| **Search number** | **Query** | **Sort By** | **Filters** | **Search Details** | **Results** | **Time** |
| --- | --- | --- | --- | --- | --- | --- |
| **4** | (("clinical trial"[Title/Abstract] OR "RCT"[Title/Abstract] OR "randomized controlled trial"[Title/Abstract] OR "clinical study"[Title/Abstract]) AND ("BERT"[Title/Abstract] OR "RoBERTa"[Title/Abstract] OR "ALBERT"[Title/Abstract] OR "Generative Pre-trained Transformer"[Title/Abstract] OR "GPT-2"[Title/Abstract] OR "GPT-3"[Title/Abstract] OR "GPT-4"[Title/Abstract] OR "GPT-3.5"[Title/Abstract] OR "ChatGPT"[Title/Abstract] OR "T5"[Title/Abstract] OR "Transformer"[Title/Abstract] OR "XLNet"[Title/Abstract] OR "ELECTRA"[Title/Abstract] OR "LLAMA"[Title/Abstract] OR "Claude"[Title/Abstract] OR "Bard"[Title/Abstract] OR "LLM"[Title/Abstract] OR "LLMs"[Title/Abstract] OR "Large language model"[Title/Abstract])) NOT ("editorial"[Publication Type] OR "comment"[Publication Type] OR "letter"[Publication Type] OR "news"[Publication Type] OR "conference abstract"[Title/Abstract] OR "brief communication"[Title/Abstract]) |  | from 2018 - 2024 | ((("clinical trial"[Title/Abstract] OR "RCT"[Title/Abstract] OR "randomized controlled trial"[Title/Abstract] OR "clinical study"[Title/Abstract]) AND ("BERT"[Title/Abstract] OR "RoBERTa"[Title/Abstract] OR "ALBERT"[Title/Abstract] OR "Generative Pre-trained Transformer"[Title/Abstract] OR "GPT-2"[Title/Abstract] OR "GPT-3"[Title/Abstract] OR "GPT-4"[Title/Abstract] OR "GPT-3.5"[Title/Abstract] OR "ChatGPT"[Title/Abstract] OR "T5"[Title/Abstract] OR "Transformer"[Title/Abstract] OR "XLNet"[Title/Abstract] OR "ELECTRA"[Title/Abstract] OR "LLAMA"[Title/Abstract] OR "Claude"[Title/Abstract] OR "Bard"[Title/Abstract] OR "LLM"[Title/Abstract] OR "LLMs"[Title/Abstract] OR "Large language model"[Title/Abstract])) NOT ("editorial"[Publication Type] OR "comment"[Publication Type] OR "letter"[Publication Type] OR "news"[Publication Type] OR "conference abstract"[Title/Abstract] OR "brief communication"[Title/Abstract])) AND (2018:2024[pdat]) | 317 | 05:58:38 |
| **3** | (("clinical trial"[Title/Abstract] OR "RCT"[Title/Abstract] OR "randomized controlled trial"[Title/Abstract] OR "clinical study"[Title/Abstract]) AND ("BERT"[Title/Abstract] OR "RoBERTa"[Title/Abstract] OR "ALBERT"[Title/Abstract] OR "Generative Pre-trained Transformer"[Title/Abstract] OR "GPT-2"[Title/Abstract] OR "GPT-3"[Title/Abstract] OR "GPT-4"[Title/Abstract] OR "GPT-3.5"[Title/Abstract] OR "ChatGPT"[Title/Abstract] OR "T5"[Title/Abstract] OR "Transformer"[Title/Abstract] OR "XLNet"[Title/Abstract] OR "ELECTRA"[Title/Abstract] OR "LLAMA"[Title/Abstract] OR "Claude"[Title/Abstract] OR "Bard"[Title/Abstract] OR "LLM"[Title/Abstract] OR "LLMs"[Title/Abstract] OR "Large language model"[Title/Abstract])) NOT ("editorial"[Publication Type] OR "comment"[Publication Type] OR "letter"[Publication Type] OR "news"[Publication Type] OR "conference abstract"[Title/Abstract] OR "brief communication"[Title/Abstract]) |  |  | (("clinical trial"[Title/Abstract] OR "RCT"[Title/Abstract] OR "randomized controlled trial"[Title/Abstract] OR "clinical study"[Title/Abstract]) AND ("BERT"[Title/Abstract] OR "RoBERTa"[Title/Abstract] OR "ALBERT"[Title/Abstract] OR "Generative Pre-trained Transformer"[Title/Abstract] OR "GPT-2"[Title/Abstract] OR "GPT-3"[Title/Abstract] OR "GPT-4"[Title/Abstract] OR "GPT-3.5"[Title/Abstract] OR "ChatGPT"[Title/Abstract] OR "T5"[Title/Abstract] OR "Transformer"[Title/Abstract] OR "XLNet"[Title/Abstract] OR "ELECTRA"[Title/Abstract] OR "LLAMA"[Title/Abstract] OR "Claude"[Title/Abstract] OR "Bard"[Title/Abstract] OR "LLM"[Title/Abstract] OR "LLMs"[Title/Abstract] OR "Large language model"[Title/Abstract])) NOT ("editorial"[Publication Type] OR "comment"[Publication Type] OR "letter"[Publication Type] OR "news"[Publication Type] OR "conference abstract"[Title/Abstract] OR "brief communication"[Title/Abstract]) | 450 | 05:58:31 |
| **2** | (("clinical trial"[Title/Abstract] OR "RCT"[Title/Abstract] OR "randomized controlled trial"[Title/Abstract] OR "clinical study"[Title/Abstract]) AND ("BERT"[Title/Abstract] OR "RoBERTa"[Title/Abstract] OR "ALBERT"[Title/Abstract] OR "Generative Pre-trained Transformer"[Title/Abstract] OR "GPT-2"[Title/Abstract] OR "GPT-3"[Title/Abstract] OR "GPT-4"[Title/Abstract] OR "GPT-3.5"[Title/Abstract] OR "ChatGPT"[Title/Abstract] OR "T5"[Title/Abstract] OR "Transformer"[Title/Abstract] OR "XLNet"[Title/Abstract] OR "ELECTRA"[Title/Abstract] OR "LLAMA"[Title/Abstract] OR "Claude"[Title/Abstract] OR "Bard"[Title/Abstract] OR "LLM"[Title/Abstract] OR "LLMs"[Title/Abstract] OR "Large language model"[Title/Abstract])) |  |  | ("clinical trial"[Title/Abstract] OR "RCT"[Title/Abstract] OR "randomized controlled trial"[Title/Abstract] OR "clinical study"[Title/Abstract]) AND ("BERT"[Title/Abstract] OR "RoBERTa"[Title/Abstract] OR "ALBERT"[Title/Abstract] OR "Generative Pre-trained Transformer"[Title/Abstract] OR "GPT-2"[Title/Abstract] OR "GPT-3"[Title/Abstract] OR "GPT-4"[Title/Abstract] OR "GPT-3.5"[Title/Abstract] OR "ChatGPT"[Title/Abstract] OR "T5"[Title/Abstract] OR "Transformer"[Title/Abstract] OR "XLNet"[Title/Abstract] OR "ELECTRA"[Title/Abstract] OR "LLAMA"[Title/Abstract] OR "Claude"[Title/Abstract] OR "Bard"[Title/Abstract] OR "LLM"[Title/Abstract] OR "LLMs"[Title/Abstract] OR "Large language model"[Title/Abstract]) | 464 | 05:56:36 |
| **1** | (("clinical trial"[Title/Abstract] OR "RCT"[Title/Abstract] OR "randomized controlled trial"[Title/Abstract] OR "clinical study"[Title/Abstract]) AND ("BERT"[Title/Abstract] OR "RoBERTa"[Title/Abstract] OR "ALBERT"[Title/Abstract] OR "Generative Pre-trained Transformer"[Title/Abstract] OR "GPT-2"[Title/Abstract] OR "GPT-3"[Title/Abstract] OR "GPT-4"[Title/Abstract] OR "GPT-3.5"[Title/Abstract] OR "ChatGPT"[Title/Abstract] OR "T5"[Title/Abstract] OR "Transformer"[Title/Abstract] OR "XLNet"[Title/Abstract] OR "ELECTRA"[Title/Abstract] OR "LLAMA"[Title/Abstract] OR "Claude"[Title/Abstract] OR "Gemini"[Title/Abstract] OR "Bard"[Title/Abstract] OR "LLM"[Title/Abstract] OR "LLMs"[Title/Abstract] OR "Large language model"[Title/Abstract])) |  |  | ("clinical trial"[Title/Abstract] OR "RCT"[Title/Abstract] OR "randomized controlled trial"[Title/Abstract] OR "clinical study"[Title/Abstract]) AND ("BERT"[Title/Abstract] OR "RoBERTa"[Title/Abstract] OR "ALBERT"[Title/Abstract] OR "Generative Pre-trained Transformer"[Title/Abstract] OR "GPT-2"[Title/Abstract] OR "GPT-3"[Title/Abstract] OR "GPT-4"[Title/Abstract] OR "GPT-3.5"[Title/Abstract] OR "ChatGPT"[Title/Abstract] OR "T5"[Title/Abstract] OR "Transformer"[Title/Abstract] OR "XLNet"[Title/Abstract] OR "ELECTRA"[Title/Abstract] OR "LLAMA"[Title/Abstract] OR "Claude"[Title/Abstract] OR "Gemini"[Title/Abstract] OR "Bard"[Title/Abstract] OR "LLM"[Title/Abstract] OR "LLMs"[Title/Abstract] OR "Large language model"[Title/Abstract]) | 500 | 05:56:18 |

**Embase**

#1 AND #2

#2

(2018:py OR 2019:py OR 2020:py OR 2021:py OR 2022:py OR 2023:py OR 2024:py) AND [embase]/lim NOT ([embase]/lim AND [medline]/lim) AND ('chatgpt'/de OR 'clinical article'/de OR 'clinical study'/de OR 'clinical trial'/de OR 'clinical trial topic'/de OR 'comparative study'/de OR 'controlled clinical trial'/de OR 'controlled study'/de OR 'double blind procedure'/de OR 'human experiment'/de OR 'large language model'/de OR 'major clinical study'/de OR 'multicenter study'/de OR 'phase 1 clinical trial'/de OR 'phase 2 clinical trial'/de OR 'phase 3 clinical trial'/de OR 'predictive model'/de OR 'randomized controlled trial'/de OR 'randomized controlled trial topic'/de OR 'single blind procedure'/de) AND 'article'/it

#1

('clinical trial':ab,ti OR 'rct':ab,ti OR 'randomized controlled trial':ab,ti OR 'clinical study':ab,ti) AND ('bert':ab,ti OR 'roberta':ab,ti OR 'albert':ab,ti OR 'generative pre-trained transformer':ab,ti OR 'gpt-2':ab,ti OR 'gpt-3':ab,ti OR 'gpt-4':ab,ti OR 'gpt-3.5':ab,ti OR 'chatgpt':ab,ti OR 'transformer':ab,ti OR 'xlnet':ab,ti OR 'electra':ab,ti OR 'llama':ab,ti OR 'claude':ab,ti OR 'bard':ab,ti OR 'llm':ab,ti OR 'llms':ab,ti OR 'large language model':ab,ti)

**Scopus**

( TITLE-ABS-KEY ( "clinical trial" OR rct OR "randomized controlled trial" OR "clinical study" ) AND TITLE-ABS-KEY ( bert OR roberta OR albert OR "Generative Pre-trained Transformer" OR gpt-2 OR gpt-3 OR gpt-4 OR "GPT-3.5" OR chatgpt OR transformer OR xlnet OR electra OR llama OR claude OR bard OR llm OR llms OR "Large language model" ) ) AND PUBYEAR > 2017 AND PUBYEAR < 2025 AND ( LIMIT-TO ( EXACTKEYWORD , "ChatGPT" ) OR LIMIT-TO ( EXACTKEYWORD , "Large Language Model" ) OR LIMIT-TO ( EXACTKEYWORD , "Randomized Controlled Trial" ) OR LIMIT-TO ( EXACTKEYWORD , "Clinical Study" ) ) AND ( EXCLUDE ( DOCTYPE , "cp" ) OR EXCLUDE ( DOCTYPE , "re" ) OR EXCLUDE ( DOCTYPE , "le" ) OR EXCLUDE ( DOCTYPE , "no" ) OR EXCLUDE ( DOCTYPE , "er" ) OR EXCLUDE ( DOCTYPE , "ed" ) OR EXCLUDE ( DOCTYPE , "sh" ) OR EXCLUDE ( DOCTYPE , "ch" ) )

**ICTRP**

""Large Language model" OR "Large Language Models" OR "LLM" OR "LLMs" OR "ChatGPT" OR "GPT-4" OR "GPT-2" OR "GPT-3" OR "GPT-3.5" OR "transformer" OR "Generative Pre-trained Transformer" OR "Claude" OR "Llama" OR "bard"
