# Supplementary material for: Large language models in medicine: A review of current clinical trials across healthcare applications
Source: PLOS Digit Health. 2024 Nov 19;3(11):e0000662. doi: 10.1371/journal.pdig.0000662 (PMC11575759; doi:10.1371/journal.pdig.0000662)
Supplement: S1 Table — (DOCX) [file pdig.0000662.s002.docx]

**S1 Table: A comprehensive summary of the included studies – running clinical trials.**

| ID | Date | Recruitment Status | Sample Size | Center Type | Country | Field, category | Model | Study Objective | Primary Outcome |
| --- | --- | --- | --- | --- | --- | --- | --- | --- | --- |
| NCT06346496 | 28/03/2024 | Not recruiting | 657 | Single | China | Mental health, Patient Care | LLM-based AI Dialogue Bot | Intervention for Depression and Anxiety Symptoms | Anxiety, Mood changes, Depression (Day before, Day 14, Day 28) |
| ChiCTR2400081938 | 15/03/2024 | Not recruiting | 1000 | Multi | China | Internal medicine, Patient Care | ChatGPT | Quality of remote diagnosis and treatment | Blood pressure compliance, Medication compliance, Patient satisfaction |
| NCT06321328 | 25/03/2024 | Recruiting | 398 | Single | Turkey | Intensive Care, Decision and Diagnostics Aid | GPT-4 | Predicting postoperative care needs | Postoperative intensive care needs and mortality |
| DRKS00033775 | 11/03/2024 | Not recruiting | 600 | Single | Germany | General, Patient Care | ChatGPT | Decision-making using symptom checkers | Accuracy in medical case assessments |
| NCT06276049 | 17/02/2024 | Not recruiting | 103 | Single | China | Medical training, Decision and Diagnostics Aid | ChatGPT | Enhancing self-directed learning | Self-Directed Learning Scale, Critical Thinking, Learning Flow |
| NCT06263855 | 01/07/2024 | Recruiting | 1015 | Single | USA | General, Data Handling | CURE | Assisting discharge summary writing | Rate of patient accrual |
| NCT06247475 | 29/01/2024 | Recruiting | 120 | Single | Taiwan | General, Decision and Diagnostics Aid | GPT-3.5, GPT-4 | Simulating virtual consultations | Correctness of answers, Satisfaction and Bloom's taxonomy correlation |
| NCT06229379 | 03/01/2024 | Recruiting | 84 | Single | China | Ophthalmology, Patient Care | Digital twin patient | Enhancing clinical questioning skills | Medical history acquisition exam scores |
| NCT06208423 | 20/12/2023 | Recruiting | 50 | Single | USA | General, Decision and Diagnostics Aid | GPT-4 | Physician reasoning on management cases | Management reasoning, Time spent on management |
| ChiCTR2300078274 | 03/12/2023 | Completed | 60 | Single | China | Orthopedics, Patient Care | CPT-4 | Informed consent in surgery | Patient satisfaction, Anxiety and Depression scales, Patient satisfaction |
| NCT06157944 | 27/11/2023 | Completed | 50 | Multi | USA | General, Decision and Diagnostics Aid | GPT-4 | Diagnostic reasoning | Diagnostic reasoning and time |
| DRKS00032895 | 18/10/2023 | Completed | 200 | Single | Germany | General, Decision and Diagnostics Aid | GPT-4 | Accuracy with patient-generated case vignettes | Accuracy in assessing medical cases |
| NCT06002425 | 13/08/2023 | Recruiting | 400 | Multi | Germany, Italy, China, USA | Gastroenterology, Decision and Diagnostics Aid | ChatGPT | Treatment recommendations for cancers | Influence of ChatGPT on treatment plans, Progression-Free Survival (PFS) |
| NCT05963802 | 04/07/2023 | Completed | 26 | Single | Canada | Medical training, Research Assistance | ChatGPT | AI usability in health sciences training | System Usability Scale, Student perception |
| NCT05945004 | 30/06/2023 | Not recruiting | 120 | Single | USA | General surgery, Data Handling | GPT-4 | Preoperative visit sheet writing | Satisfaction for clinical use, Familiarity to human writing |
| NCT06009783 | 19/06/2023 | Recruiting | 40 | Single | Canada | Urology, Patient Care | ChatGPT | Pre-vasectomy counselling | Consultation length, Patient satisfaction, Number of questions asked |
