# Supplementary material for: Large language models in medicine: A review of current clinical trials across healthcare applications
Source: PLOS Digit Health. 2024 Nov 19;3(11):e0000662. doi: 10.1371/journal.pdig.0000662 (PMC11575759; doi:10.1371/journal.pdig.0000662)
Supplement: S2 Table — (DOCX) [file pdig.0000662.s003.docx]

**S2 Table: A comprehensive summary of the included studies – published clinical trials.**

| Author et al. | Year | Sample Size | Center Type | Country | Field, Category | Model | Objective | Primary Outcome | Results | Authors' Conclusion |
| --- | --- | --- | --- | --- | --- | --- | --- | --- | --- | --- |
| Baker et al. | 2024 | 11 | One center | USA | General, Data Handling | GPT-4 | Investigated ChatGPT's ability to assist in writing a history of present illness | Documentation quality | ChatGPT produced longer and higher quality patient histories, had intermediate documentation speeds, and included erroneous information in 36% of documents | ChatGPT has potential to improve clinical documentation but concerns remain about accuracy, privacy, and bias |
| Civettini et al. | 2024 | 6 | Multi-center | Italy | Hematology, Decision and Diagnostics Aid | GPT-4, PaLm 2, Llama-2 13b, and Llama-2 70b | Assessed HSCT decision-making by providing decisions on transplant eligibility, donor selection, conditioning regimens, and transplant-related mortality | Overall agreement and kappa values comparing responses of LLMs and residents with the expert consensus | LLMs showed moderate agreement with expert consensus but were generally outperformed by medical residents | LLMs demonstrated promising capabilities that could support clinicians in complex medical decisions with further refinement |
| Deveci et al. | 2023 | 36 | One center | Denmark | Medical research, Research Assistance | GPT-4 | Application of ChatGPT-4 model to write cover letters for scientific paper submissions | Impression, readability, criteria satisfaction, and detail of cover letters | No significant differences in impression, readability, and detail between GPT-4 and human-written cover letters; GPT-4's cover letters were more readable | GPT-4 is non-inferior to humans in writing cover letters for scientific journals, suggesting it can streamline the cover letter writing process |
| Lawrence et al. | 2024 | 40 | One center | USA | Medical research (Orthopedics), Research Assistance | GPT-3 | Used ChatGPT to generate abstracts for arthroplasty literature, evaluated against human-written abstracts for perceived quality and authorship | Confidence in authorship source and rated effectiveness of communication and overall quality of the abstracts | Reviewers had higher confidence in human authorship for human-written abstracts; AI-generated abstracts achieved comparable scores in authorship discernibility | Human-written abstracts were preferred slightly over AI-generated ones, but AI presented a considerable challenge in authorship discernibility |
| Bitar et al. | 2022 | 386 | Multi-center | Saudi Arabia | Gynecology, Patient Care | BERT | Used BERT to generate summarized text about HPV to educate participants, compared with full-length text | HPV knowledge through a questionnaire | Women who read the original text showed slightly better understanding in some areas of HPV knowledge compared to those who read the BERT-generated summarized text | Using BERT for text summarization was promising in increasing women's knowledge about HPV, making educational material more accessible and time-efficient |
